# Changes in potassium and sodium concentrations in stored blood

**DOI:** 10.11604/pamj.2015.20.236.5851

**Published:** 2015-03-12

**Authors:** Clement Opoku-Okrah, Benjamin Kojo Safo Acquah, Elliot Eli Dogbe

**Affiliations:** 1 Department of Medical Laboratory Technology, Kwame Nkrumah University of Science and Technology, Kumasi, Ghana; 2 Transfusion Medicine Unit, Komfo Anokye Teaching Hospital, Kumasi, Ghana

**Keywords:** Potassium concentration, sodium concentration, stored blood, hyperkalaemia and hyponatraemia, Ghana

## Abstract

Potassium is the principal intracellular cation with sodium being the principal extracellular cation. Maintenance of the distribution of potassium and sodium between the intracellular and the extracellular compartments relies on several homeostatic mechanisms. This study analysed the effect of blood storage on the concentrations of potassium and sodium in stored blood and also determine any variations that may exist in their concentrations. 50mls of blood was sampled each from 28 units of evenly mixed donated blood in citrate phosphate dextrose adenine (CPDA-1) bags immediately after donation into satellite bag and stored at 4oC. Potassium and sodium concentration determinations were done on each of the 28 samples on day 0 (before blood was initially stored in the fridge), day 5, day 10, day 15 and day 20 of storage using the Roche 9180 ISE Electrolyte Analyser (Hoffmann-La Roche Ltd, Switzerland). data analysis showed significant changes in the potassium and sodium concentrations with a continuous rise in potassium and a continuous fall in sodium. A daily change of 0.59mmol/l and 0.50mmol/l was observed in the potassium and sodium concentrations respectively. We showed steady but increased daily concentrations of potassium and decrease concentrations of sodium in blood stored over time at 4oC.

## Introduction

Electrolyte disturbances can be associated with a number of occurrences including drug usage [1] but the kidney is expected to manage it. Hypokalaemia and hyperkalaemia has been seen as problem for some hospitalized patients [2] with hyperkalaemia being implicated for complications of massive blood transfusion [3–5]. It has been observed that following blood transfusion of stored blood, complications such as hyperkalaemia, hyponatraemia and citrate toxicity among other conditions do occur [6]. During blood storage, there is a slow but constant leakage of potassium from the cells into the surrounding plasma along a concentration gradient as a result of sodium potassium ATPase pump failure. The plasma level of potassium may increase by 0.5-1.0mmol/L per day of refrigerator storage [7]. There is a notion that the total amount of extracellular potassium in a unit of blood stored for 35days falls within 7mmol/L to 25mmol/L [8]. The resultant impact of transfusion of stored blood on the potassium and acid-base balance on the recipient is very complex. It is however, largely dependent on the rate of transfusion, volume of blood transfused, the rate of citrate metabolism and the changing state of the peripheral perfusion of the patient/recipient [3, 9]. Failure to establish the apparent electrolyte changes has been found to be fatal in some instances [10].

Sodium is the major electrolyte in the extracellular fluid (ECF) with about 98% of its totality being in the ECF and only 2% being in the intracellular fluid (ICF) with a reference range of 135-145mEq/L. Sodium levels below135mEq/L results in a condition termed hyponatraemia caused by low levels of sodium or excess water in relation to the amount of sodium with post-operative patients commonly experiencing it [11]. A drop in sodium concentration causes cellular oedema which affects the central nervous system and leads to depression and cerebral oedema [12]. Sodium levels above 145mEq/L results in hypernatremia, a condition that is generally associated with a hyperosmolar state where a fluid volume exists. The increase in extracellular sodium causes intracellular to shift out of the cells into extracellular spaces, which results in cellular dehydration. Also, cardiac output is reduced due to decreased myocardial contractility leading to heart failure [13]. Potassium is the major element in the intracellular fluid (ICF) with about 98% of its totality being the ICF leaving 2% in the ECF with a reference range of 3.5-5.0mEq/L; thus plasma potassium being of a small fraction of, and an accurate reflection of whole body potassium [14, 15]. Potassium storage and its distribution within the body has been noted to be regulated by very important hormones with its normal transcellular distribution of high intracellular to extracellular ratio being maintained by three hormonal signals, insulin, aldosterone and β-adrenergic catecholamines [16]; buffering of ECF potassium through uptake of potassium by cell is greatly impaired by the absence of aldosterone, insulin or catecholamines [17]. Potassium uptake by all cells is through the Na-K ATPase pump; being one of the most permeable ions across cell membranes and makes use of the potassium channels to exit cells: in some cells via K-H exchange or via K-Cl co-transport) [18]. Potassium remains the major ion determinant of the resting membrane electrical potential, which limits and opposes potassium efflux giving rise to changes in ECF potassium concentrations that have marked effects on cell excitability within the heart, brain, nerve and muscles [19]. ICF potassium leakage to the ECF increases when osmolarity increases, as in diabetes mellitus and in metabolic acidosis during which it is exchanged for ECF protons (H+). High potassium content of cell is release upon cell death leading to the increase in ECF potassium concentration [15, 20–22]. Potassium plays an important role in cellular metabolism, especially in protein and glycogen synthesis and in the enzymatic processes necessary for cellular energy. A constant cell potassium concentration is critical for enzyme activities and for cell division and growth. It also aids in maintaining cellular electrical neutrality and osmolality.

Other functions of potassium include acid-base balance, nerve impulse conduction and maintenance of normal cardiac rhythm as well as skeletal and smooth muscle contraction [14]. Conditions where serum potassium levels are below 3.5mEq/L is termed hypokalaemia and can be found in 20% hospitalized patients [23]. Potassium may also be lost through kidney excretion in association with metabolic alkalosis and hyperaldosteronism [22, 24]. Potassium levels below 3.0mEq/L can cause a problem with cardiovascular and neuromuscular function causing compromised respiratory function [25]. Hypokalaemia also results in glucose intolerance by depressing insulin release from the pancreas [16, 17]. Cardiac and / or respiratory arrest can also result from very low levels of potassium [26]. Hyperkalaemia is a condition where the serum potassium level is above 5.0mEq/L and is most often related to kidney failure due to inadequate kidney function [24]. Hyperkalaemia could lead to fatal arrhythmias if not diagnosed and managed early enough [27]. The aim of this study was therefore to analysis the daily changes in sodium and potassium concentrations in stored blood at 4oC over a 20-day period at a teaching hospital blood bank in Ghana.

## Methods

We used 28 donated samples obtained at the Komfo Anokye Teaching Hospital, Kumasi-Ghana that had been screened using the protocol of the Transfusion Medicine Unit of the hospital. The bleeding was done using CPDA-1 double bags after which 50mls of the thoroughly mixed blood and anticoagulant was transferred into the satellite bag for storage at 4oC for use in the study; enabling the rest to be used for transfusion purposes. We did 5 electrolyte concentration determinations on each of the 28 samples on day 0 (before blood was initially stored in the fridge), day 5, day 10, day 15 and day 20 of storage. On the respective day of analysis, 2 mls of the blood sample was placed into a gel tube and spun at 1500rpm for 3minutes to obtain the plasma. The plasma was then analyzed using the Roche 9180 ISE Electrolyte Analyser (Hoffmann-La Roche Ltd, Switzerland).

## Results

**Figure 1:**
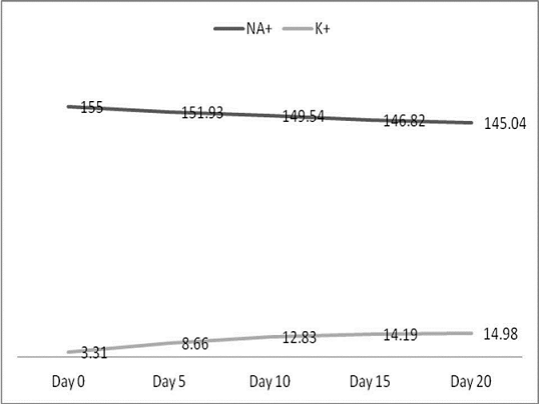
Mean measurement of electrolyte concentration per reading

Figure 1 shows the mean sodium and potassium levels of all the samples in relation to the various days’ readings. It shows a gradual consistent reduction in sodium concentration having reduced from a mean of 155mmol/l to 145.04mmol/l, a change of approximately 10mmol/l over the 20-day period. The potassium levels on the other hand shot up sharply from 3.31mmol/l on day 0 to 8.66mmol/l on day 5 and further to 12.83mmol/l on day 10 after which the rise seemed to have slowed reading 14.19mmol/l on day 15 and 14.98mmol/l on day 20. Table 1 shows the mean changes in the electrolyte concentration over the 20-day period. There is a daily change in potassium concentration of 1.08mmol/l during the first five days of storage; day 6 to day 10 recorded a daily potassium change of 0.84mmol/l; day 11 to day 15 recorded a 0.28mmol/l daily change whiles day 16 to day 20 recorded daily change 0.16mmol/l giving an overall average daily potassium change of 0.59mmol/l over the 20-day period. In terms of the sodium concentration, there was a change of 0.61mmol/l during the first five days of storage; day 6 to day 10 recorded a daily sodium change of 0.48mmol/l; day 11 to day 15 recorded a 0.54mmol/l daily change whiles day 16 to day 20 recorded daily change 0.36mmol/l giving an overall average daily sodium change of 0.50mmol/l over the 20-day period.

**Table 1:** Bonferroni's multiple comparison test of variance between the various reading days

DAY	POTASSIUM			SODIUM		
	Mean Diff.	*t*-value	*p*-value	Mean Diff.	*t*-value	*p*-value
DAY 0 vs DAY 5	-5.4	19	<0.05	3.071	4.030	<0.05
DAY 0 vs DAY 10	-9.5	33	<0.05	5.464	7.169	<0.05
DAY 0 vs DAY 15	-11	38	<0.05	8.179	10.73	<0.05
DAY 0 vs DAY 20	-12	41	<0.05	9.964	13.07	<0.05
DAY 5 vs DAY 10	-4.2	15	<0.05	2.393	3.140	<0.05
DAY 5 vs DAY 15	-5.5	19	<0.05	5.107	6.701	<0.05
DAY 5 vs DAY 20	-6.3	22	<0.05	6.893	9.044	<0.05
DAY 10 vs DAY 15	-1.4	4.8	<0.05	2.714	3.561	<0.05
DAY 10 vs DAY 20	-2.1	7.5	<0.05	4.500	5.904	<0.05
DAY 15 vs DAY 20	-0.79	2.8	>0.05	1.786	2.343	>0.05

## Discussion

Sodium as the primary ECF cation is expected to have a reference range of 145-155mmol/l plasma concentration. This project saw a reduction in ECF sodium concentration in line with work done by Wallas (1979, 2010). The mean total sodium concentration over the period was seen to have dropped from a level of 155mmol/l to a level of 145.04mmol/l (Figure 1). This drop is of great significance and concern due to the numeric variation occurring between readings (Table 1). It is observed that there was a change of 0.61mmol/l during the first five days of storage; day 6 to day 10 recorded a daily sodium change of 0.48mmol/l; day 11 to day 15 recorded a 0.54mmol/l daily change whiles day 16 to day 20 recorded daily change 0.36mmol/l giving an overall average daily sodium change of 0.50mmol/l over the 20-day period. This average daily change is likely to move sodium concentrations in stored blood to level outside the normal reference range as it is stored over time and this holds possible adverse clinical effect on recipients of such units. This is likely to increase the chances of such recipient being prone to oedema as collaborated by Metheny [28] especially in patients with low sodium intake or those experiencing diarrhoea. Potassium on the other hand being mainly an ICF cation is expected to have an ECF concentration of 3.5-5.5mmol/l [14, 15]. The average potassium concentration for this study from Day 0 through Day 20 (Figure 1) were 3.31mmol/l, 8.66mmol/l, 12.73mmol/l, 14.19mmol/l and 14.98mmol/l showing a steep rise in in potassium concentration in consonance with established work [29, 30]. There is a daily change in potassium concentration of 1.08mmol/l during the first five days of storage; day 6 to day 10 recorded a daily potassium change of 0.84mmol/l; day 11 to day 15 recorded a 0.28mmol/l daily change whiles day 16 to day 20 recorded daily change 0.16mmol/l giving an overall average daily potassium change of 0.59mmol/l over the 20-day period. The seemingly sharp drop in the daily change between days 11 and 15 as well as days 16 and 20 can be attributed to the averaging of the readings at 15mmol/l for most of the readings done in the last days as a result of the limitation on the analyser's reading ability. It was realized that the analyser was unable to read potassium concentrations above 15mmol/l leading to all readings above 15mmol/l being truncated at 15mmol/l.

However, there was relatively higher increment in potassium concentrations on day 10 and 15 by 21.4% and 60.7% respectively. The increased in potassium concentrations over the 20-day measurement period correlated with other works by Latham et al., [31] and Ratcliffe et al., [8] who also gave increased concentrations above 25mmol/l during a 21-35 storage period. Concurrently, the average daily change of 0.59mmol/l gotten from this research duly falls in line with work done by Bailey and Bove [7] who stated that plasma level of potassium may increase by 0.5-1.0mmol/L per day of refrigeration. The general decrease in ECF sodium concentration and increase in ECF potassium concentration will also result a concomitant opposite effect in the ICF where sodium and potassium concentrations also changes due to the failure of the sodium-potassium pump. The failure of the Na+/K+ ATPase action during the storage period was first postulated by Wallas [32]; he attributed the failure to an inhibition of the membrane ATPase by the low temperature allowing the unopposed leakage of these electrolytes in and out of the red cells. Normally, the Na+/K+ pump is known to transport 3 sodium ions against 2 potassium ions [33] but from our study, a careful look at the daily changes in the sodium concentration (0.5mmol/l) and potassium changes (0.59mmol/l) shows an almost equal exchange of the two electrolytes between the ECF and ICF as blood is stored in the fridge at 4oC. We can therefore deduce that though there is a failure in the ATPase activity, the resultant occurrence is closely regulated by the sodium - potassium pump.

## Conclusion

Due to the increase in potassium level with storage, care is needed in determining age of blood [34], volume transfused at a time and rate of transfusion [35] to minimize hyperkalaemia related blood transfusion complications [4, 35, 36]. Transfusing hyperkalaemic blood however, may be a transient factor if the recipient's kidneys are functioning properly [2] and the Na+/K+ ATPase is also working efficiently [18, 37]. However, this is a great concern in individuals undergoing dialysis [14] due to renal failure.
